# Supplementary material for: How Well Do Rodent Models of Parkinson’s Disease Recapitulate Early Non-Motor Phenotypes? A Systematic Review
Source: Biomedicines. 2022 Nov 24;10(12):3026. doi: 10.3390/biomedicines10123026 (PMC9775565; doi:10.3390/biomedicines10123026)
Supplement: Supplementary file 1 [file biomedicines-10-03026-s001.zip › biomedicines-2032191-supplementary.pdf]

Supplementary Materials

Supplementary Table S1

Study characteristics, phenotypic outcomes, and tests performed in genetic PD models used in 3 studies or greater.

Ref No.	Author, year	Animal model	Rodent	Background strain	Promoter	Sex	Age (months)	Outcome	Test Performed	Motor or Cell Count tested?
[1]	Griffioen et al, 2013	**A53T**	Mouse	B6;C3H	Thy1	M	2, 5, 7-11	↓ Cardio	ECG telemetry	NA
[2]	Kuo et al, 2010	**A53T**<br>PAC crossed with SNCA KO	Mouse	FVB/N 129S6/SvEvTac	Endogenous gene regulatory elements	MF	3-18	Olfaction, — 12mo<br><br>GI function, ↓ 3-18mo<br><br>Cardio, — 12mo	Non-Social Novel Odour Test, Buried Food Test<br><br>Faecal and water content, Bead expulsion test, Whole gut transit time<br><br>ECG telemetry	Motor, ↓ 6-18mo
[3]	Carboni et al, 2017	**A53T**	Mouse	B6;C3H	PrnP	NS	7	↓ Cognition	Novel Object Recognition	↓ Motor<br><br>— Cell Count
[4]	Valek et al, 2021	**A53T**<br>(Study tested A53T*PINK1 KO but not A53T for olfaction)	Mouse	FVB/N	PrnP	NS	9	NA	NA	— Motor
[5]	Kuo et al, 2019	**A53T**<br>PAC crossed with SNCA KO	Mouse	129S6/SvEvTac	Endogenous gene regulatory elements	M	4, 7, 9	↓ GI function	Bead Expulsion Test, Whole Gut Transit Time	Motor, —7mo
[6]	Seo et al, 2020	**A53T**	Mouse	C57BL/6J	PrnP	NS	8, 10	↓ Olfaction	Buried Pellet Test	— Motor
[7]	Farrell et al, 2014	**A53T**	Mouse	C57BL/6J	PrnP	NS	12-14	↓ Anxiety<br><br>↓ Olfaction	Centre of Open Field, Hyponeophagia Test<br>Buried Pellet Test	NA
[8]	Wi et al, 2018	**A53T**	Mouse	C57BL/6J	PrnP	NS	8, 10	↓ Olfaction	Buried Pellet Test	NA

[9]	Zhang et al, 2015	**A53T**	Mouse	B6/C3H	PrnP	M	3, 6, 10	Olfaction,<br>— 3mo<br>↓ 6, 10mo	Block Test (social discrimination),<br>Habituation Dishabituation,<br>Buried Food Test	Motor,<br>— 10mo<br><br>Cell Count,<br>— 10mo
[10]	Gerson et al, 2018	**A53T**	Mouse	B6/C3H	PrnP	MF	7	↓ Cognition	Novel Object Recognition	— Motor
[11]	Diwakarla et al, 2021	**A53T**	Mouse	B6/C3H	PrnP	MF	15-19	GI function,<br>↓ 16-19mo	Bead Expulsion Test,<br>Whole Gut Transit Time	Motor,<br>↓ 19mo
[12]	Diwakarla et al, 2020	**A53T**	Mouse	B6/C3H	PrnP	MF	5-15	GI function,<br>— 5-14mo<br>↓ 15mo	Bead Expulsion Test,<br>Whole Gut Transit Time,<br>Faecal and Water Content	Motor,<br>↓ 8-15mo
[13]	Taguchi et al, 2020	**A53T**<br>BAC	Mouse	C57Bl/6	Endogenous gene regulatory elements	MF	3-18	Circadian<br>↓ 11, 18mo<br><br>Olfaction,<br>↓ 9mo<br><br>Anxiety,<br>— 9mo<br><br>Cognition,<br>— 9mo	Sleep Recordings<br><br><br>Urine Odour Test,<br>Olfactory Avoidance Test<br><br>Light Dark Box<br><br><br>Spatial Recognition<br>Barnes Maze	Motor,<br>— 9mo<br><br>Cell Count,<br>— 3mo<br>↓ 9, 18mo
[14]	Li et al, 2021	**A53T**	Mouse	C57Bl/6	NS	MF	20	↓ Cognition<br><br>↓ Olfaction	Fear Conditioning<br><br>Olfactory Bedding Test	↓ Motor<br><br>↓ Cell Count
[15]	Do et al, 2021	**A53T**	Mouse	C57BL/6	PrnP	MF	8, 10, 12, 14, 16 monthly to 23	Cognition,<br>— 8-23mo<br><br>Olfaction,<br>— 8-23mo	Novel Object Recognition<br><br><br>Buried Pellet Test	Motor,<br>↓ 8-23mo
[16]	Stanojlovic et al, 2019	**A53T**	Mouse	C57BL/6	NS	M	3, 5, 7	↓ Cognition	Barnes Maze,<br>Contextual Object Recognition	NA
[17]	Graham et al, 2010	**A53T**	Mouse	B6/C3H	PrnP	NS	2, 8, 12	Anxiety,<br>↓ 2mo<br>— 8mo<br>↑ 12mo	Elevated Plus Maze	Motor,<br>↓ 2mo<br>— 8mo<br>↑ 12mo
[18]	Costa et al, 2020	**A53T**	Mouse	B6/C3H	PrnP	MF	12	↓ Olfaction<br>↓ GI function	Buried Pellet Test<br>Faecal Pellet Output	—Motor<br>↓ Cell Count

								↓ Cognition	Novel Object Recognition, Spontaneous Alternation	
[19]	Peters et al, 2020	**A53T**	Mouse	C57Bl/6	PrnP	MF	3-4, 6-7, 9-10	Circadian, ↓ 3-10mo	EMG/EEG Recordings	NA
[20]	Paumier et al, 2013	**A53T**	Mouse	C57BL/6	PrnP	M	2, 6, 12	Cognition, — 2mo ↓ 6, 12mo<br><br>Anxiety, — 2mo ↑ 6, 12mo	Spatial Recognition<br><br>Centre of Open Field	Motor, ↑ 2, 6, 12mo
[21]	Thom et al, 2022	**A53T** crossed with cellular Prp KO	Mouse	C57Bl/6	PrnP	M	9, 18	Anxiety, ↓ 9, 18mo	Elevated Plus Maze	Motor, — 18mo
[22]	Singh et al, 2019	**A53T**	Mouse	C57Bl/6	PrnP	M	6, 12	↓ Cognition	Barnes Maze, Spatial Recognition Fear Conditioning	↑ Motor
[23]	Pfeffer et al, 2018	**A53T**	Mouse	FVB/N	PrnP	M	4-6	↓ Circadian	Re-entrainment test	— Motor
[24]	Zhang et al, 2018	**A53T**	Mouse	NS	PrnP	M	12	↓ Cognition	Novel Object Recognition, Spatial Recognition	↓ Motor
[25]	Rockenstein et al, 2016	**A53T**	Mouse	C57Bl/6	PrnP	NS	2	↓ Cognition<br><br>— Anxiety	Fear Conditioning<br><br>Centre of Open Field	↑ Motor
[26]	West et al, 2020	**A53T**	Mouse	C57BL/6	PrnP	M	7	↓ GI Function	In vitro colonic monitoring, Faecal Pellet Output	NA
[27]	Noorian et al, 2012	**A53T**	Mouse	FVB/N	PrnP	MF	2-3, 8-10, 14-16, 18-20	GI Function, — 2, 9mo ↓ 15-20mo	Faecal Pellet Output, Gastric Emptying	NA
[28]	Tikhonova et al, 2020	**A53T**	Mouse	C57Bl/6	PrnP	M	6-8	↓ Cognition	Passive Avoidance Test, Spontaneous Alternation, Barnes Maze	↑ Motor
[29]	Wang et al, 2018	**A53T**	Mouse	B6/C3H	PrnP	M	3, 6, 9, 12	GI Function, ↓ 3, 6, 9mo ↑12mo<br><br>Depression, — 3mo ↓ 6mo<br>↓ Anxiety, 3, 6mo	Faecal Pellet Output<br><br>Tail Suspension Test<br><br>Centre of Open Field	Motor, ↑ 3, 6 mo — 9, 12 mo

[30]	Li et al, 2020	**A53T**	Mouse	B6/C3H	PrnP	M	3, 6, 9, 12	Anxiety,<br>— 3mo<br>↓ 6, 9, 12mo<br><br>Depression,<br>— 3mo<br>↓ 6, 9, 12mo	Elevated Plus Maze<br><br><br><br>Tail Suspension Test,<br>Forced Swim Test	Motor,<br>— 3, 6, 9mo<br>↓ 12mo
[31]	Rota et al, 2019	**A53T**	Mouse	C57Bl/6	PrnP	MF	3, 6, 9, 12, 14	GI function,<br>↓ 3-12mo	Whole Gut Transit Time,<br>Faecal Pellet Output	Motor,<br>— 3, 6mo
[32]	Kim et al, 2014	**A53T**	Mouse	NS	PrnP	NS	14-15	↓ Anxiety	Light Dark Box,<br>Elevated Plus Maze	— Motor
[33]	Vidal-Martinez et al, 2016	**A53T**	Mouse	B6/C3H	PrnP	NS	1-15	GI function,<br>— 1-5mo<br>↓ 6-15mo	Faecal Pellet Output,<br>Whole Gut Transit Time	NA
[34]	Huang et al, 2019	**A53T**	Mouse	NS	PrnP	NS	12	↓ Cognition	Spatial Recognition	↓ Motor
[35]	Finkelstein et al, 2016	**A53T**	Mouse	C57BL/6	PrnP	NS	8	↓ Cognition	Morris Water Maze,<br>Spatial Recognition<br>Novel Object Recognition	↓ Motor<br><br>↓ Cell Count
[36]	Oaks et al, 2013	**A53T**	Mouse	B6/C3H	PrnP	NS	2, 4, 8, 12	Depression,<br>— 2, 12mo<br>↑ 4-8mo<br><br>Anxiety,<br>— 2, 4, 8mo<br>↑ 12mo	Forced Swim Test<br><br><br><br>Elevated Plus Maze	Motor,<br>↓ 2-12mo
[37]	Sardi et al, 2017	**A53T**	Mouse	B6/C3H	PrnP	NS	4, 8	↓ Cognition,<br>4, 8mo	Novel Object Recognition,<br>Fear Conditioning	NA
[38]	Wu et al, 2016	**A53T**	Mouse	B6/C3H	PrnP	MF	7	— Depression<br><br>— Olfaction	Sucrose Preference Test<br><br>Buried Pellet Test	↓ Motor
[39]	Rothman et al, 2013	**A53T**	Mouse	C57Bl/6	Thy1	M	3-7	Circadian,<br>↓ 3mo<br><br>Anxiety,<br>↑ 3-6mo	Circadian Monitoring System<br><br>Elevated Plus Maze	Motor,<br>↓ 3-7mo
[40]	Tatenhorst et al, 2016	**A53T**	Mouse	B6	PrnP	NS	6, motor impairm ent onset	Cognition,<br>— 6mo<br>↓ ~16mo	Novel Object Recognition	Motor,<br>— 1-6mo<br>↓ ~16mo
[25]	Rockenstein et al, 2016	**Ha-syn OE**<br>Thy1-asyn	Mouse	C57Bl/6-DBA/2	Thy1	F	6, 9	Anxiety,<br>↑ 6, 9mo	Centre of Open Field	Motor,<br>↑ 6, 9mo

								Olfaction,<br>↓9mo	Buried Pellet Test	
[41]	Hallett et al, 2012	**Ha-syn OE**<br>Thy1-asyn	Mouse	C57BL/6-DBA/2	Thy1	M	2-4, 12-15	GI Function,<br>— 2-4mo<br>↓ 9-12	Faecal Pellet Output,<br>Whole Gut Transit Time	Motor,<br>↓ 12-15mo
[42]	Yamakado et al, 2012	**Ha-syn OE**<br>a-syn BAC	Mouse	NS	Endogenous gene regulatory elements	NS	12-13, 24	Cognition,<br>— 12, 24mo<br><br>Anxiety,<br>↑13mo,<br>— 24mo	Radial Maze Test,<br>Barnes Maze Test,<br>Fear Conditioning<br><br>Elevated Plus Maze,<br>Centre of Open Field	Motor,<br>↑ 13mo<br><br>Cell Count,<br>— 24mo
[43]	Mandler et al, 2014	**Ha-syn OE**<br>1) Thy1-asyn<br>2) PDGF-asyn	Mouse	NS	Thy1,<br>PDGF- β	NS	9-10 (Thy-1 asyn),<br>12 (PDGF-asyn)	Cognition,<br>↓ 12 mo<br>(PDGF-asyn)	Morris Water Maze	Motor,<br>↓ 9-12 (Thy1-asyn)
[44]	Wassouf et al, 2018	**Ha-syn OE**<br>Asyn BAC	Mouse	C57BL/6	Endogenous gene regulatory elements	MF	6	Anxiety,<br>↓ 6mo	Light Dark Box,<br>Elevated Plus Maze	Motor,<br>— 6mo<br>↓ 13, 20mo
[45]	Subramaniam et al, 2018	**Ha-syn OE**<br>Thy1-asyn	Mouse	C57BL6/DBA/2	Thy1	M	2, 4, 8	Cognition,<br>— 5mo<br>↓ 6-7mo	Spontaneous Alternation<br>Novel Object Recognition	Motor,<br>↓ 2, 4, 6, 8mo
[46]	Brown et al, 2021	**Ha-syn OE**<br>Thy1-asyn	Mouse	BDF-1	Thy1	M	1, 3	Olfaction,<br>↓ 1mo<br>— 3mo	Buried Pellet Test	Motor,<br>↓ 1, 3mo<br><br>Cell Count,<br>— 3mo
[47]	Games et al, 2014	**Ha-syn OE**<br>Thy1-asyn	Mouse	C57Bl/6-DBA/2	Thy1	F	12	↓ Cognition	Morris Water Maze	↓ Motor<br><br>— Cell Count
[48]	Schwab et al, 2017	**Ha-syn OE**<br>H-asynL62	Mouse	C57Bl/6	Thy1	MF	5.5	↓ Anxiety	Light Dark Box	NA
[49]	Wang et al, 2012	**Ha-syn OE**<br>Thy1-asyn	Mouse	C57BL/6-DBA/2	Thy1	M	2-5, 7-10, 12-14, 18	GI Function,<br>↓ 2-3, 7-8 mo<br>— 4-5, 9-10, 12-14, 18mo	Faecal Pellet Output,<br>Gastric Emptying	Motor,<br>↑ 4-5mo

								Anxiety,<br>↑ 4-5mo	Centre of Open Field	
[50]	McDowell et al, 2014	**Ha-syn OE** Thy1-asyn	Mouse	C57BL/6-DBA/2	Thy1	M	9.5-10.5	↓ Circadian	EEG/EMG Recordings	NA
[51]	Bermudez et al, 2019	**Ha-syn OE** Thy1-asyn	Mouse	BDF1	Thy1	M	2, 4	Olfaction,<br>↓ 2mo<br>— 4mo	Buried Pellet Test	Motor,<br>↓ 2, 4mo
[52]	Biju et al, 2020	**Ha-syn OE** Thy1-asyn	Mouse	C57Bl/6	Thy1	M	18	↓ Olfaction	Buried Pellet Test	↓ Motor
[53]	Fleming et al, 2008	**Ha-syn OE** Thy1-asyn	Mouse	C57BL/6-DBA/2	Thy1	M	3-4, 5-6, 9, 11	↓ Olfaction	Buried Pellet Test,<br>Block Test,<br>Habituation/Dishabituation	NA
[54]	Fleming et al, 2011	**Ha-syn OE** Thy1-asyn	Mouse	C57BL/6-DBA/2	Thy1	M	3-4	↓ Olfaction	Buried Pellet Test	↓ Motor
[55]	Fleming et al, 2013	**Ha-syn OE** Thy1-asyn	Mouse	C57BL/6-DBA/2	Thy1	M	3-5, 9-12	↓ Cardio	Telemetry	NA
[56]	Magen et al, 2012	**Ha-syn OE** Thy1-asyn	Mouse	C57BL/6-DBA/2	Thy1	M	3-9, 11-13	Cognition,<br>— 3, 11-13mo<br>↓ 5-9mo	Spontaneous Alternation,<br>One-trial Object-Place Recognition,<br>Novel Object Recognition,<br>Operant Learning Task	NA
[57]	Torres et al, 2021	**Ha-syn OE** Thy1-asyn	Mouse	C57Bl/6-DBA/2	Thy1	M	3-4	↑ Cognition<br><br>— Anxiety	Fear Conditioning<br><br>Centre of Open Field	— Motor
[21]	Thom et al, 2022	**Ha-syn OE** Thy1-asyn	Mouse	C57Bl/6	Thy1	M	9	↓ Cognition<br><br><br>↑ Anxiety	Novel Object Recognition,<br>Fear conditioning<br><br>Elevated Plus Maze	— Motor
[58]	Wang et al, 2008	**Ha-syn OE** Thy1-asyn	Mouse	C57Bl/6-DBA/2	Thy1	M	11-12	↓ GI Function	Bead Expulsion Test, Faecal Pellet Output	NA
[59]	Li et al, 2022	**Ha-syn OE** Thy1-asyn	Mouse	C57Bl/6	Thy1	M	6, 7, 9, 12	Olfaction,<br>↓ 6, 7, 9, 12mo<br><br>Depression,<br>— 7mo	Buried Pellet Test,<br>Block Test<br><br><br>Sucrose Preference Test	Motor,<br>— 6, 9mo<br>↓ 12mo<br><br>Cell Count<br>↓ 12mo
[60]	Grant et al, 2014	**Ha-syn OE** Thy1-asyn	Mouse	C57BL/6-DBA/2	Thy1	M	2-3, 6-7, 9	↓ Cognition	USV Recordings	NA
[61]	Keane et al, 2019	**A30P**	Mouse	C57Bl/6	NS	MF	3, 13	Cognition,<br>— 13mo	Barnes Maze	Motor,<br>— 3-12mo

										Cell Count, ↓13mo
[2]	Kuo et al, 2010	**A30P** PAC crossed with SNCA KO	Mouse	NS	Endogenous gene regulatory elements	MF	6-12	GI function, ↓3-18mo	Faecal Pellet Output, Bead Expulsion Test, Whole Gut Transit Time	Motor, — 6, 12mo
[62]	Gureviciene et al, 2009	**A30P**	Mouse	C57BL/6	PrnP	M	7-9, 22-25	Cognition, — 7-9, 22-24mo	Morris Water Maze	Motor, — 7-9mo, ↓22-24mo
[5]	Kuo et al, 2019	**A30P** PAC crossed with SNCA KO	Mouse	29S6/SvEvTac_FVB/N_C57/BL6	Endogenous gene regulatory elements	M	4, 7, 9	↓GI Function	Bead Expulsion	— Motor
[22]	Singh et al, 2019	**A30P**	Mouse	C57Bl/6	PrnP	M	12	— Cognition	Spatial Recognition	NA
[63]	Marxreiter et al, 2013	**A30P** Tet regulated	Mouse	NS	CaMK	M	2	↓Anxiety	TMT-induced Fear, Elevated Plus Test	— Motor
[64]	Schell et al, 2012	**A30P**	Mouse	NS	Thy1	M	6-18	Cognition, ↓6, 16mo	Fear Conditioning	NA
[65]	Neuner et al, 2014	**A30P**	Mouse	C57Bl/6	Thy1	NS	6-8	Olfaction, ↓6mo<br><br>Cognition, — 8mo	Olfactory Discrimination Test (with cognitive component)	NA
[66]	Freichel et al, 2007	**A30P**	Mouse	C57Bl/6	Thy1	NS	4, 9-15, 16-18	Cognition, — 4mo ↓12mo	Morris Water Maze, Fear Conditioning, Active Avoidance Test	Motor, — 4, 9-15mo ↑12mo ↓16-18mo
[67]	Stylianou et al, 2020	**A30P**	Mouse	C57Bl/6	Thy1	M	2.5-4	↓Circadian	In vivo recordings in PFC, CA1 hip	NA
[68]	Gries et al, 2021	**A30P**	Mouse	C57Bl/6	Thy1	MF	2, 12-13	GI Function, ↓2mo	GI motility recording in organ bath	Motor, —2mo ↓12-13mo
[69]	Langley et al, 2021	**Mitopark**	Mouse	NS	Dopamine Transporter (DAT)	MF	1-5	Olfaction, — 1-2mo ↓3-5mo<br><br>Cognition, — 1-2mo ↓3-5mo	Social and Novel Scent Test<br><br>Morris Water Maze	Motor, — 1-2mo ↓3-5mo

								Depression,<br>— 1-2mo<br>↓ 3-5mo<br><br>Anxiety,<br>— 1-2mo<br>↓ 3-5mo<br><br>Circadian,<br>— 1-5mo	Force Swim Test,<br>Tail Suspension Test<br><br>Elevated Plus Maze<br><br>Sleep Latency Test	
[70]	Li et al, 2013	**Mitopark**	Mouse	C57Bl/6	DAT	MF	1-5	Cognition,<br>↓ 1mo	Barnes Maze,<br>Novel Object Recognition	Motor,<br>— 1-2mo<br>↓ 3-5mo
[71]	Cong et al, 2016	**Mitopark**	Mouse	C57Bl/6	DAT	MF	5-7	Cognition,<br>↓ 5-6mo<br><br>Depression<br>↓ 5-6mo	Novel Object Recognition<br><br>Tail Suspension Test	Motor,<br>↓ 7mo
[72]	Ghaisas et al, 2019	**Mitopark**	Mouse	C57BL/6	DAT	MF	2-5	GI Function,<br>↓ 3-5mo	Gastric Emptying,<br>Whole Gut Transit Time,<br>Bead Expulsion Test	NA
[73]	Fifel et al, 2014	**Mitopark**	Mouse	NS	DAT	NS	2-6	Circadian,<br>— 2mo<br>↓ 6mo	Re-entrainment Test	NA
[74]	Paβ et al, 2020	**Mitopark**	Mouse	C57Bl/6	DAT	MF	1-4, 6	Olfaction,<br>—1-3,<br>↓ 4, 6mo	Buried Pellet Test,<br>Odour Discrimination	Cell Count,<br>↓ 4-6mo
[75]	Langley et al, 2018	**Mitopark**	Mouse	C57Bl/6	DAT	MF	2	— Olfaction	Social Odour Discrimination	↓ Motor
[76]	Baumann et al, 2016	**VMAT2 KO**	Mouse	C47Bl/6	NS	M	12	↓ Depression	Saccharin Preference Test,<br>Forced Swim Test	NA
[77]	Cui et al, 2021	**VMAT2 KO**	Mouse	NS	Dual promoters (*Phox2a*, *Ph ox2b*, *Hand 2* or *Gata3* f rom mice)	MF	12, 18	↓ Cognition	Morris Water Maze	↓ Motor
[78]	Taylor et al, 2009	**VMAT2 KO**	Mouse	C57Bl/6	NS	MF	2, 4, 5, 6, 12, 18	Olfaction,<br>— 2-4mo<br>↓ 5-12mo	Social and Non-Social Olfactory Discrimination Test	Motor,<br>— 2, 18mo

								Anxiety,<br>↓ 2mo<br>— 18mo<br><br>Depression,<br>— 2, 18mo<br><br>Circadian,<br>↑ 2mo<br>— 18mo<br><br>GI function,<br>↓ 2, 18mo	Elevated Plus Maze<br><br>Forced Swim Test,<br>Tail Suspension Test<br><br>Sleep Latency Test<br><br>Gastric Emptying	
[79]	Rabl et al, 2017	**Ha-syn OE (Thy1-asyn) hemi**	Mouse	NA	C57Bl/6xDBA		1-8	Cognition,<br>— 2-3, 6mo<br>↓ 8mo	Fear Conditioning	Motor,<br>— 1mo<br>↑ 2-3, 6mo
[80]	Gabreilyan et al, 2021	**Ha-syn OE (Thy1-asyn) hemi**	Mouse	NA	C57Bl/6xDBA2	MF	5-6	Anxiety,<br>↓ 6mo<br><br>Olfaction,<br>↓ 6mo	Marble Burying Test<br><br>Buried Pellet Test	Motor,<br>↓ 5mo
[81]	Goldberg et al, 2015	**Ha-syn OE (Thy1-asyn) hemi**	Mouse	NA	C57Bl/6	NS	13	↓ Cognition	Novel Object Recognition, Object Place Recognition	↓ Motor
[82]	Lim et al, 2018	**LRRK2 G2019S** Human gene	Mouse	B6;C3H	PDGFB	MF	2-4, 9-11, 14-19	Anxiety,<br>— 2-4mo,<br>↓ 9-11, 14-19mo<br><br>↓ Depression	Light Dark Box,<br>Elevated Plus Maze<br><br>Sucrose Preference Test,<br>Forced Swim Test,<br>Tail Suspension Test	Motor,<br>— 2-4, 9-11mo<br>↓ 14-19mo
[83]	Matikainen-Ankney et al, 2018	**LRRK2 G2019S** Mouse gene	Mouse	C57BL/6NTac	Endogenous gene regulatory elements	M	2	— Anxiety<br><br>— Depression	Elevated Plus Maze<br><br>Sucrose Preference Test	— Motor
[84]	Volta et al, 2015	**LRRK2 G2019S** BAC human gene	Mouse	C57Bl/6	Human regulator	M	3, 6, 12	Cognition,<br>— 3mo,<br>↓ 12mo	Novel Object Location,<br>Novel Object Recognition	Motor,<br>— 6, 12mo
[85]	Crown et al,	**LRRK2**	Mouse	C57BL/6NTa	Endogenou	M	8-10	↓ Circadian	Sleep Recording	— Motor

	2020	**G2019S**<br>Mouse gene		c	s gene regulatory elements					
[86]	Adeosun et al, 2017	**LRRK2 G2019S**	Mouse	C57Bl/6	NS	M	8-9	↓ Cognition	Radial Arm Water Maze, Spatial Recognition, Spontaneous Alternation	— Motor
[4]	Valek et al, 2021	**PINK1 KO**<br>(Study tested A53T*PINK1 KO but not PINK1 KO for olfaction)	Mouse	129/SvEv; FVB/N	NA	NS	9	NA	NA	— Motor
[87]	Glasl et al, 2012	**PINK1 KO**	Mouse	C57Bl/6	NA	MF	3-9, 24-27	Cognition,<br>— 4mo<br><br>Olfaction,<br>↓ 27mo	Novel Object Recognition<br><br>Olfactory Discrimination Test with Reward Associations	Motor,<br>— 3-4, 24-26mo<br><br>Cell Count,<br>— 6, 19mo
[88]	Maynard et al, 2020	**PINK1 KO**	Mouse	C57Bl/6	NA	M	9-11	↓ Cognition<br><br>— Anxiety	Spontaneous Alternation, Fear conditioning<br><br>Marble Burying	— Motor
[89]	Agnihotri et al, 2019	**PINK1 KO**	Mouse	C57Bl/6	NA	MF	3	— Depression<br><br>— Anxiety	Tail Suspension Test, Forced Swim Test, Sucrose Preference Test<br><br>Novelty Suppressed Feeding Test,<br>Elevated Plus Maze	— Motor
[90]	Ferris et al, 2018	**PINK1 KO**	Rat	Long Evans	NA	M	2	— Cognition	Novel Object Recognition, Barnes Maze	↓ Motor
[91]	Hoffmeister et al, 2021	**PINK1 KO**	Rat	Long Evans	NA	M	4, 8, 12	↓ Anxiety	Elevated Plus Maze	NA
[92]	Marquis et al, 2020	**PINK1 KO**	Rat	Long Evans	NA	F	2, 4, 6, 8	↓ Depression<br><br>↓ Anxiety	Sucrose Preference Test<br><br>Elevated Plus Maze, Light/Dark Box	↓ Motor
[93]	Billia et al, 2011	**PINK1 KO**	Mice	NA	NA	M	2, 6	↓ Cardio	ECG Recordings	NA
[19]	Peters et al, 2020	**Tau KO**	Mouse	C57Bl/6	NA	MF	3-4, 6-7, 9-10	— Circadian	EMG/EEG recordings	NA

[94]	Beauchamp et al, 2018	**Tau KO**	Mouse	Sv129B/6	NA	MF	7, 12, 15	Olfaction,<br>↓ 7, 12mo<br>—15mo	Non-Social Olfactory Discrimination Test	Motor,<br>— 7, 12mo<br>↓ 15mo
[95]	Li et al, 2014	**Tau KO**	Mouse	C57Bl/6	NA	MF	22-24	— Cognition	Spontaneous Alternation, Fear Conditioning	— Motor
[96]	Lei et al, 2012	**Tau KO**	Mouse	NS	NA	NS	6, 12, 24	Cognition,<br>— 6,12mo	Spontaneous Alternation	Motor,<br>— 6mo,<br>↓ 12-24mo<br><br>Cell Count,<br>— 6mo<br>↓ 12mo
[97]	Lei et al, 2014	**Tau KO**	Mouse	Compared two background strains: Bl6/129sv and C57Bl/6	NA	MF	12	Cognition,<br>↓ for Bl6/129sv,<br>— for C57Bl/6	Spatial Recognition	↓ Motor<br>Both models<br><br>↓ Cell Count<br>Both models
[98]	Morris et al, 2013	**Tau KO**	Mouse	C57Bl/6	NA	MF	12-15, 21-22	— Cognition	Novel Object Recognition, Morris Water Maze	Motor,<br>↓ 12-15mo<br>— 21-22mo
[22]	Singh et al, 2019	**Tau KO**	Mouse	C57Bl/6	NA	M	6, 12	— Cognition	Fear Conditioning, Barnes Maze	— Motor
[99]	Dongworth et al, 2014	**DJ-1 KO**	Mouse	C57Bl/6	NA	NS	NS	— Cardio	ECG Recordings	NA
[100]	Pham et al, 2010	**DJ-1 KO**	Mouse	C57Bl/6	NA	MF	6-7, 13-14	Cognition,<br>— 6-7mo<br>↓ 13-14mo	Novel Object Recognition	NA
[101]	Li et al, 2019	**DJ-1 KO**	Mouse	B6;129	NA	M	3-5	↓ Anxiety	Elevated Plus Maze, Light Dark Box	↓ Motor
[102]	Billia et al, 2013	**DJ-1 KO**	Mouse	NA	NA	MF	2	— Cardio	ECG Recordings	NA
[103]	Kyser et al, 2019	**DJ-1 KO**	Rat	Long Evans	NA	M	2, 4, 7, 12, 16	Cognition,<br>— 4mo<br><br>Anxiety,<br>— 4mo<br><br>Depression,<br>↓ 6mo<br>— 15mo	Novel Object Recognition<br><br>Elevated Plus Maze<br><br>Forced Swim Test, Sucrose Preference Test	Motor,<br>— 2mo<br>↓ 4, 7mo

								Olfaction,<br>— 16mo	Buried Pellet Test	
[104]	Liu et al, 2018	**A53T Hemi**	Mouse	B6/C3H	PrnP	NS	3, 6, 9, 12	Cognition,<br>↑ 3, 9mo	Morris Water Maze	Motor,<br>— 3, 6, 9mo<br>↑12mo
[105]	Uemura et al, 2021	**A53T Het**<br>BAC	Mouse	C57Bl/6	Endogenous gene regulatory elements	M	4, 9-12	Olfaction,<br>— 4mo<br><br>Anxiety,<br>— 9mo<br><br>Depression,<br>— 9, 11mo<br><br>Cognition,<br>— 9, 10<br><br>Circadian,<br>— 12mo	Social and Novel Odour Discrimination<br><br>Light Dark Box<br><br>Forced Swim Test,<br>Tail Suspension Test<br><br>Spontaneous Alternation,<br>Barnes Maze,<br>Fear Conditioning<br><br>Home Cage Activity	Motor,<br>— 9mo
[106]	Hamill et al, 2012	**A53T Hemi**	Mouse	B6/C3H	PrnP	MF	1-16	Urinary,<br>↓ 4-16mo	Cystometric Recordings	Motor,<br>↓ 11-16mo
[107]	La Vitola et al, 2021	**A53T Hemi**	Mouse	C57Bl/6	PrnP	NS	8	— Cognition	Novel Object Recognition,<br>Spontaneous Alternation,<br>Morris Water Maze	↑ Motor<br><br>↓ Cell Count
[108]	Tikhonova et al, 2020	**A53T Hemi**	Mouse	C57Bl/6	PrnP	M	6	↓ Cognition	Passive Avoidance Test	↑ Motor
[109]	Giesert et al, 2017	**LRRK2 R1441C**	Mouse	C57BL/6	NS	NS	2, 3, 5, 24-26	Olfaction,<br>↓ 24-26mo<br><br>Cognition,<br>— 3mo<br><br>Depression,<br>↓ 7-8mo	Olfactory Discrimination Test with Reward Association<br><br>Novel Object Recognition<br><br>Forced Swim Test,<br>Tail Suspension Test	Motor,<br>— 3mo<br>↓ 24-26mo<br><br>Cell Count,<br>— 5, 28 mo
[110]	Bichler et al, 2013	**LRRK2 R1441G**<br>BAC	Mouse	FVB/N	human LRRK2 promoter/enhancer regions on the BAC	NS	4, 6, 9, 12, 14, 16, 18-21	Anxiety,<br>— 6, 14, 19mo<br><br>Depression,	Elevated Plus Maze<br><br>Tail Suspension Test,	Motor,<br>— 2-12mo<br>↓16-21mo

					transgene			— 6-19mo<br><br>Cognition, — 21mo<br><br>GI function, ↓ 6, 9, 21mo<br><br>Olfaction, — 6, 14mo	Forced Swim Test<br><br>Passive Avoidance Test<br><br>Faecal Pellet Output<br><br>Block Test, Buried Pellet Test	
[111]	Mikhail et al 2015	**LRRK2 R1441G** BAC	Mouse	FVB	NS	NS	6-9	— Cognition	Fear Conditioning	— Motor
[112]	Dranka et al, 2014	**LRRK2 R1441G** BAC	Mouse	FVB/N	human LRRK2 promoter/enhancer regions on the BAC transgene	M	15	↓ Olfaction	Buried Pellet Test	↓ Motor
[113]	Lopatina et al, 2014	**CD157 KO**	Mouse	C57Bl/6	NA	M	2	↓Anxiety<br><br>↓Depression<br><br>↑ Cognition<br><br>— Circadian	Centre of Open Field, Light Dark Box, Elevated Plus Maze<br><br>Tail Suspension Test Forced swim Test<br><br>Fear Conditioning<br><br>Home Cage Activity	— Motor
[114]	Mizuno et al, 2015	**CD157 KO**	Mouse	C57Bl/6	NA	M	NS	↓ Anxiety	Anxiety and Social Preference Test with Social Target	NA
[115]	Kasai et al, 2017	**CD157 KO**	Mouse	C57Bl/6	NA	M	2	↓ Anxiety<br><br>↓ Depression	Centre of Open Field<br><br>Forced Swim Test	↓ Motor
[116]	Higashida et al, 2017	**CD157 KO**	Mouse	C57Bl/6	NA	MF	1	↓ Anxiety	Light Dark Box	NA
[117]	Maekawa et al, 2019	**LRRK2 KO**	Mouse	C57Bl/6	NA	M	1-3	— GI Function	Bead Expulsion Test, Intestinal Transit Assay	NA
[118]	Beccano-Kelly et al, 2015	**LRRK2 KO**	Mouse	NSA	NA	M	3-6	— Cognition	Novel Object Recognition	— Motor

[119]	Maset et al, 2021	**LRRK2 KO**	Mouse	C57Bl/6	NA	MF	1	↓ Olfaction	Habituation/Dishabituation Test	NA
[84]	Volta et al, 2015	**LRRK2 KO**	Mouse	C57Bl/6	NA	M	12	— Cognition	Novel Object Location, Novel Object Recognition	— Motor
[109]	Giesert et al, 2017	**LRRK2 KO**	Mouse	NS	Human U6	NS	3-4, 11-13, 24	Olfaction, — 24mo<br><br>Cognition, — 4mo<br><br>Depression, ↓ 11-13mo	Olfactory Discrimination Test with Reward Association<br><br>Novel Object Recognition<br><br>Forced Swim Test, Tail Suspension Test	Motor, — 3, 24mo
[120]	Navarro et al, 2008	**Parkin KO** Exon 2 deletion	Mouse	C57BL/6	NA	M	6-8, 15-17	↓ Cognition	Novel Object Recognition	NA
[121]	Zhu et al, 2007	**Parkin KO** Exon 3 deletion	Mouse	C57Bl/6	NA	NS	6, 12, 15, 18, 21	Cognition, ↓ No age<br><br>Anxiety, ↓ 6-15, 21mo	Morris Water Maze<br><br>Light Dark Box	— Motor
[122]	Rial et al, 2014	**Parkin KO** Exon 3 deletion	Mouse	C57Bl/6	NA	M	5-6	— Olfaction<br><br>— Depression<br><br>— Anxiety<br><br>↓ Cognition	Olfactory Bedding Discrimination Test<br><br>Forced Swim Test, Tail Suspension Test<br><br>Elevated Plus Maze<br><br>Novel Object Recognition, Spatial Recognition	↑ Motor
[123]	Itier et al, 2003	**Parkin KO** Exon 3 deletion	Mouse	129SV/C57BL6	NA	MF	2-24	Cognition, ↓ 4mo	Spontaneous Alternation	Motor, ↓ 5-6, 12mo<br><br>Cell Count, — 15mo
[124]	Perez et al, 2005	**Parkin KO** Exon 2 deletion	Mouse	B6;129S4	NA	M	3, 6, 12, 18-22	Depression, — 12 ↓ 20mo<br><br>Anxiety, —12, 20m	Forced Swim Test, Tail Suspension Test<br><br>Light Dark Box, Elevated Plus Maze	↓ Motor

								Cognition,<br>↑ 12mo<br>—21mo	Novel Object Recognition,<br>Spontaneous Alternation,<br>Straight-alley-swim escape,<br>Black-White-Discrimination Swim task,<br>Lashley III Swim Maze,<br>Morris Water Maze,<br>Passive Avoidance Test	
[125]	Kubli, et al 2013	**Parkin KO**	Mouse	129S4/SvJae	NA	M	3	—Cardio	ECG Recordings	NA
[126]	Hollville et al, 2020	**Parkin KO**<br>Exon 3 deletion	Mouse	C57Bl/6	NA	MF	3-7, 17-19mo	Olfaction,<br>– 6-7mo<br><br>Cognition,<br>– 5-7mo	Buried Pellet Test<br><br>Morris Water Maze	Motor,<br>– 3-5mo<br><br>Cell Count,<br>– 17-19mo

Table 1. Summary of all animal model characteristics, phenotypic tests performed and the outcomes. ↓ represents a significant impairment, ↑ represents an improvement and — represents no change between wildtype and transgenic animals. M – male, F – female, MF – male and female, NS – not specified. BAC – Bacterial Artificial Chromosomes, PAC – P1 derived artificial chromosome.

Supplementary Table S2

Study characteristics, phenotypic outcomes, and tests performed in genetic PD models used in 2 studies or fewer.

Ref no.	Author, Year	Model	Rodent	Target	Background Strain	Sex and Age (mo)	Outcome	Test Performed	Motor or Cell Count?
[127]	Pavia-Collado et al, 2021	A30P/A53T	Mouse	Double transgenic model of alpha-synuclein mutations under TH promotor	C57BL/6J	M,<br>5	↓ Anxiety<br><br>– Depression<br><br>↓ Cognition	Light Dark Box<br><br>Tail Suspension Test<br><br>Novel Object Recognition	↓ Motor<br><br>– Cell Count
[128]	Lelan et al, 2011	A30P/A53T	Rat	Double transgenic model of A30P and A53T under TH promotor	Sprague Dawley	F,<br>6, 9, 14, 18	Olfaction,<br>↓ 6, 9, 14mo	Olfactory Discrimination Test	Motor,<br>– 14, 18mo
[4]	Valek et al, 2021	PINK KO/A53T	Mouse	Double transgenic model containing PINK KO and A53T	129/SvEv; FVB/N	NS,<br>13-16	Olfaction,<br>– 16mo	NOR using odours	Motor,<br>– 13-16mo

				mutation					
[15]	Do et al, 2021	GBA+/-/A53T and GBA+/-	Mouse	Double transgenic of GBA+/- and A53T, and model of single GBA+/- mutation	C57BL/6	MF,<br>8, 10, 12, 14, then monthly to 23mo	— Cognition<br>Both models<br><br>Olfaction,<br>↓ 20mo<br>Both models	NOR<br><br><br>Buried Pellet Test	— Motor<br>Both models
[22]	Singh et al, 2019	Tau KO/A53T	Mouse	Double transgenic with mutant A53T and lacking endogenous Tau	C57BL/6	M,<br>3, 6, 12	— Cognition	Fear Conditioning,<br>Y Maze	Motor,<br>↑ 6, 12mo
[19]	Peters et al, 2020	Tau KO/A53T	Mouse	Double transgenic with mutant A53T and lacking endogenous Tau	C57BL/6	MF,<br>3-4, 6-7, 9-10	↓ Circadian	EMG/EEG recordings	NA
[129]	Ikuno et al, 2019	a-syn/GBA+/-	Mouse	Double transgenic with BAC a-syn OE and heterozygous GBA mutation	B6/J	M,<br>15-18	Anxiety,<br>↑15mo<br><br>Cognition,<br>— 15mo	Centre of Open Field<br><br><br>Barnes Maze	Motor,<br>↑ 15mo<br><br>Cell count,<br>↓ 16-18mo
[130]	Hosford et al, 2020	αβγ-syn triple KO	Mouse	Triple transgenic model of alpha, beta, and gamma synuclein knock out	C57Bl/6	M,<br>3-4, 18-20	Cardio,<br>3-4mo<br>↓ 18-20mo	Non-invasive tail cuff plethysmography	NA
[131]	Tian et al, 2021	a-syn N103	Mouse	New transgenic model of PD overexpressing a-syn 1-103 fragments with Thy-1 promotor	C57BL/6	MF,<br>3, 9, 16	Anxiety,<br>— 3mo<br>↓ 9-16mo<br><br>↓ GI Function	Centre of Open Field<br><br><br><br>Faecal Pellet Output	Motor,<br>— 3mo<br>↓ 9, 16mo<br><br>Cell Count,<br>— 3mo<br>↓9,16mo
[132]	Martinez ernandez et al, 2021	Ha-syn^{119} and ha-syn^{TP}	Mouse	C-terminal truncations of haSyn (ha-syn^{119}), and a-syn gene variant with three alanine to proline substitutions at residues 30, 56 and 76 (ha-syn^{TP})	C57BL/6	M,<br>12, 18	Olfaction,<br>↓ 18mo<br>Both models	Buried Pellet Test	Motor,<br>↓ 12mo<br>Ha-syn^{119}<br>— 12mo<br>Ha-syn^{TP}<br>↓18mo<br>Both Models<br><br>— Cell Count<br>Both models
[133]	Escobar et	*SNCA*^{S129A}PAC	Mouse	2 models of S129	FVB/N x	MF,	↓ GI Function	Bead Expulsion Test	NA

	al, 2014	and *SNCA*^{S129D}PAC		phosphorylation mutation in alpha-synuclein	129S6	6	Both models		
[120]	Navarro et al, 2008	Park-/Tau^{VLW} and Tau^{VLW}	Mouse	Double transgenic model of Parkin deficient and a Tau^{VLW} model and a single model of Tau^{VLW} mutation	C57Bl/6	M,<br>6-8, 15-17	Cognition,<br>↓6-8mo<br>Both models<br>↓15-17mo<br>Tau^{VLW} only	Novel Object Recognition	NA
[111]	Mikhail et al, 2015	LRRK2 R1441G/Tau P301S and TauP301S	Mouse	Double transgenic model of LRRK2 R1441G and TauP301S mutation and a single model of TauP301S mutation	Unspecified	NS,<br>6-9	— Cognition<br>Both models	Fear Conditioning	— Motor<br>Both models
[134]	Craven et al, 2018	TauP301L	Mouse	Tau mutation model	C57Bl/6	MF,<br>5.5-7	↓Cognition<br><br>↓Circadian Rhythm	Morris Water Maze, Fear Conditioning and Extinction<br><br>Activity Onset Running Wheel Activity	↑Motor
[98]	Morris et al, 2013	Tau+/-	Mouse	Heterozygous Tau KO model	C57Bl/6	MF,<br>12-15, 21-22	Cognition,<br>—21-22mo	Novel Object Recognition, Morris Water Maze	↓Motor
[135]	Lambourne et al, 2007	TauV337M hemi	Mouse	Hemizygous mutation model of TauV337M under Thy1 promotor	C57BL/6J x CBA/Ca	M,<br>6, 10, 24-26	Cognition,<br>— 10mo<br>↓24mo	5 Choice Serial Reaction Time Test	Motor,<br>— 6, 24mo
[136]	Lambourne et al, 2005	TauV337M hemi	Mouse	Hemizygous mutation model of TauV337M under Thy1 promotor	C57Bl/6 x CBA/Ca	NS,<br>6, 12	Olfaction,<br>— 6mo<br>— 12mo<br><br>Cognition,<br>— 6mo<br>↓12mo	Food Preference Test<br><br>STFP Olfactory Memory Paradigm	Motor,<br>—12mo
[137]	Anvret et al, 2012	Adh1-/- and Adh1/4-/-	Mouse	Double KO of the ADH gene, relevant to the DA system	C57Bl/6	MF,<br>7, 15	— Olfaction<br>Both models	Olfactory Hole Poke Test	Motor,<br>↑7mo<br>Adh1 KO and Adh1/4 KO<br>↑15mo<br>Adh1 KO

									− 15mo<br>Adh1/4 KO
[138]	Belin et al, 2011	Adh4-/-	Mouse	KO of the Class IV ADH gene, relevant to the DA system	C57Bl/6	M,<br>12-17	↓ Olfaction	Olfactory Hole Poke Test	− Motor
[118]	Beccano-Kelly et al, 2015	LRRK2 OE	Mouse	Overexpression of LRRK2	Unspecified	M,<br>3-6	− Cognition	Novel Object Recognition	↓ Motor
[84]	Volta et al, 2015	LRRK2 OE	Mouse	Overexpression of LRRK2	C57Bl/6	M,<br>12	↓ Cognition	Novel Object Recognition	↓ Motor
[139]	Veenit et al, 2021	GPR37 KO	Mouse	KO of the G-protein-coupled receptor, implicated in DA system	C7Bl/6	MF,<br>3-4	↓ Anxiety<br><br>↓ Depression<br><br>↑ Cognition	Elevated Plus Maze, Light Dark Box<br><br>Forced Swim Test<br><br>Passive Avoidance Test	NA
[140]	Mandillo et al, 2013	GPR37 KO	Mouse	KO of the G-protein-coupled receptor, implicated in DA system		MF,<br>4-6, 16-18	Olfaction,<br>− 4-6mo<br>↓ 16-18mo<br><br>GI function,<br>↓ 4-6mo<br>− 16-18mo<br><br>− Cognition<br><br>Anxiety,<br>− 4-6mo<br>↓ 16-18mo<br><br>Depression,<br>− 4-6mo<br>↓ 16-18mo	Buried Pellet Test, Block Test, Odour habituation Test<br><br>Faecal Output Test<br><br>Fear conditioning<br><br>Elevated Plus Maze, Centre of Open Field, Light Dark Box<br><br>Forced Swim Test	NA
[141]	Morgan et al, 2015	DAT:TH KO and DAT-DTR	Mouse	Inactivation of Th gene in DAT neurons (DAT:TH KO), and targeted expression of human DT-receptor (DAT-DTR)	C57Bl/6	MF,<br>3-5	↓ Cognition<br>Both models	Turn and Cued Discrimination Learning,<br>Morris Water Maze,<br>Novel Object Recognition	↓ Motor<br>Both models

[142]	Buhusi et al, 2016	GDNF deficient	Mouse	Heterozygous model of GDNF deficiency; involved in DA regulation	C57Bl/6	M,<br>6-8	— Cognition	Temporal Discounting Paradigm	NA
[143]	Prediger et al, 2011	Mdk-/-	Mouse	Deletion of heparin-binding growth factor midkine, implicated in nigrostriatal development	C57Bl/6	M,<br>3-4	↓ Olfaction<br><br>↓ Cognition	Olfactory Discrimination Task<br><br>Short Term Recognition Memory Task	— Motor<br><br>↓ Cell Count
[144]	Jiang et al, 2020	VMAT2 Het	Mouse	Mice from crossing CreERT2 and floxed Vmat2 gene. Selective inactivation of one allele of VMAT2 in DA neurons.	C57Bl/6	M,<br>18	↓ Olfaction<br><br>— Circadian<br><br>— Anxiety<br><br>— Cognition<br><br>— Depression	Social and Non-Social Olfactory Discrimination Test<br><br>E-motion Test<br><br>Elevated Plus Maze<br><br>Novel Object Recognition<br><br>Sucrose Preference Test,<br>Tail Suspension Test	↓ Motor
[145]	Sonnier et al, 2007	En1+/-	Mouse	Heterozygous model lacking one En1 (engrailed1) allele, involved in DA neuron survival	Swiss	M,<br>1-11	Depression,<br>↓ 6, 8mo	Forced Swim Test, Saccharin Preference Test	Motor,<br>↓ 5mo<br>— 6mo<br><br>Cell Count,<br>— 1mo<br>↓ 2, 3, 5, 11mo
[146]	Wu et al, 2020	B4gaInt1 KO	Mouse	Disruption of *B4gaInt1* gene leading to partial deficiency of GM1 family of gangliosides	C57Bl/6	MF,<br>8-9	↓ Cognition<br><br>↓ GI Function	Alternation Task (T maze)<br><br>Faecal Pellet Output	↓ Motor<br><br>↓ Cell Count
[147]	Gil-Tommee et al, 2019	B4gaInt1 KO	Mouse	Disruption of *B4gaInt1* gene leading to partial deficiency of GM1 family of	NA	MF,<br>9, 12, 16, 19-20	Urinary,<br>—9mo<br>↓ 12-20mo	Urination Patterns, Bladder Volumes	— Motor

				gangliosides					
[148]	Parrella et al, 2019	c-rel-/-	Mouse	Model deficient for the NF-κB/c-Rel protein, a transcription factor for mitochondrial antioxidant factors	C57Bl/6	M,<br>2, 5, 9, 15, 20	↓ GI Function<br><br>— Anxiety<br><br>↓ Olfaction<br><br>— Cognition	Faecal Pellet Output<br><br>Centre of Open Field Test<br><br>Odour Detection Test,<br>Odour and item discrimination test,<br>Odour preference Test<br><br>Novel Object Recognition	NA
[126]	Hollville et al, 2020	Cul9/parkin KO and Cul9 KO	Mouse	Double knockout model of Cul9 and Parkin, both E3 ligases involved in mitochondrial function, and single Cul9 KO model	C57Bl/6	MF,<br>3-7	— Olfaction Both models<br><br>— Cognition Both models	Buried Pellet Test<br><br>Morris Water Maze	— Motor Both models<br><br>— Cell Count Both models
[149]	Ageta-Ishihara et al, 2013	SEPT4+/-	Mouse	Parkin substrate that is a component of Lewy bodies	C57Bl/6	M,<br>5-9	↑ Anxiety<br><br>— Depression<br><br>— Cognition<br><br>— Circadian	Elevated Plus Maze,<br>Light Dark Box<br><br>Forced Swim Test,<br>Tail Suspension Test<br><br>Fear Conditioning<br><br>Home Cage Monitoring of Locomotion 24hrs	— Motor
[150]	Havrda et al, 2013	Id2 KO	Mouse	Transcription factor expressed in developing central nervous system	C57Bl/6	MF,<br>1, 3, 6	Anxiety,<br>↑ 1, 3mo<br>— 6mo	Centre of Open Field,<br>Elevated Plus Maze	Motor,<br>↑ 1, 3mo<br>— 6mo<br><br>Cell Count,<br>— 1 mo<br>↓ 6mo

Table 2. Summary of all animal model characteristics, behavioural tests performed and the outcomes. ↓ represents a significant impairment, ↑ represents an improvement and – represents no change between wildtype and transgenic animals. M – male, F – female, MF – male and female, NS – not specified. PAC – P1 derived artificial chromosome.

1. Griffioen, K.J., et al., *Dietary energy intake modifies brainstem autonomic dysfunction caused by mutant alpha-synuclein.* Neurobiol Aging, 2013. **34**(3): p. 928-35.
2. Kuo, Y.M., et al., *Extensive enteric nervous system abnormalities in mice transgenic for artificial chromosomes containing Parkinson disease-associated alpha-synuclein gene mutations precede central nervous system changes.* Hum Mol Genet, 2010. **19**(9): p. 1633-50.
3. Carboni, E., et al., *Deferiprone Rescues Behavioral Deficits Induced by Mild Iron Exposure in a Mouse Model of Alpha-Synuclein Aggregation.* Neuromolecular Med, 2017. **19**(2-3): p. 309-321.
4. Valek, L., et al., *Prodromal sensory neuropathy in Pink1(-/-) SNCA(A53T) double mutant Parkinson mice.* Neuropathol Appl Neurobiol, 2021. **47**(7): p. 1060-1079.
5. Kuo, Y.M., et al., *Translational inhibition of α-synuclein by Posiphen normalizes distal colon motility in transgenic Parkinson mice.* Am J Neurodegener Dis, 2019. **8**(1): p. 1-15.
6. Seo, J.H., et al., *Environmental Enrichment Attenuates Oxidative Stress and Alters Detoxifying Enzymes in an A53T alpha-Synuclein Transgenic Mouse Model of Parkinson's Disease.* Antioxidants (Basel), 2020. **9**(10).
7. Farrell, K.F., et al., *Non-motor parkinsonian pathology in aging A53T alpha-synuclein mice is associated with progressive synucleinopathy and altered enzymatic function.* J Neurochem, 2014. **128**(4): p. 536-46.
8. Wi, S., et al., *An Enriched Environment Ameliorates Oxidative Stress and Olfactory Dysfunction in Parkinson's Disease with alpha-Synucleinopathy.* Cell Transplant, 2018. **27**(5): p. 831-839.
9. Zhang, S., Q. Xiao, and W. Le, *Olfactory dysfunction and neurotransmitter disturbance in olfactory bulb of transgenic mice expressing human A53T mutant alpha-synuclein.* PLoS One, 2015. **10**(3): p. e0119928.
10. Gerson, J.E., et al., *Tau oligomers mediate alpha-synuclein toxicity and can be targeted by immunotherapy.* Mol Neurodegener, 2018. **13**(1): p. 13.
11. Diwakarla, S., et al., *ATH434 Reverses Colorectal Dysfunction in the A53T Mouse Model of Parkinson's Disease.* J Parkinsons Dis, 2021. **11**(4): p. 1821-1832.
12. Diwakarla, S., et al., *Chronic isolation stress is associated with increased colonic and motor symptoms in the A53T mouse model of Parkinson's disease.* Neurogastroenterol Motil, 2020. **32**(3): p. e13755.
13. Taguchi, T., et al., *alpha-Synuclein BAC transgenic mice exhibit RBD-like behaviour and hyposmia: a prodromal Parkinson's disease model.* Brain, 2020. **143**(1): p. 249-265.

14. Li, H., et al., *Dl-3-n-Butylphthalide Alleviates Behavioral and Cognitive Symptoms Via Modulating Mitochondrial Dynamics in the A53T-alpha-Synuclein Mouse Model of Parkinson's Disease.* Front Neurosci, 2021. **15**: p. 647266.
15. Do, J., et al., *Behavioral Phenotyping in a Murine Model of GBA1-Associated Parkinson Disease.* Int J Mol Sci, 2021. **22**(13).
16. Stanojlovic, M., et al., *Pharmacological and chemogenetic orexin/hypocretin intervention ameliorates Hipp-dependent memory impairment in the A53T mice model of Parkinson's disease.* Mol Brain, 2019. **12**(1): p. 87.
17. Graham, D.R. and A. Sidhu, *Mice expressing the A53T mutant form of human alpha-synuclein exhibit hyperactivity and reduced anxiety-like behavior.* J Neurosci Res, 2010. **88**(8): p. 1777-83.
18. Costa, G., et al., *Gender Differences in Neurodegeneration, Neuroinflammation and Na(+)-Ca(2+) Exchangers in the Female A53T Transgenic Mouse Model of Parkinson's Disease.* Front Aging Neurosci, 2020. **12**: p. 118.
19. Peters, S.T., et al., *Ablating Tau Reduces Hyperexcitability and Moderates Electroencephalographic Slowing in Transgenic Mice Expressing A53T Human alpha-Synuclein.* Front Neurol, 2020. **11**: p. 563.
20. Paumier, K.L., et al., *Behavioral characterization of A53T mice reveals early and late stage deficits related to Parkinson's disease.* PLoS One, 2013. **8**(8): p. e70274.
21. Thom, T., et al., *Cellular Prion Protein Mediates alpha-Synuclein Uptake, Localization, and Toxicity In Vitro and In Vivo.* Mov Disord, 2022. **37**(1): p. 39-51.
22. Singh, B., et al., *Tau is required for progressive synaptic and memory deficits in a transgenic mouse model of alpha-synucleinopathy.* Acta Neuropathol, 2019. **138**(4): p. 551-574.
23. Pfeffer, M., et al., *Impaired Photic Entrainment of Spontaneous Locomotor Activity in Mice Overexpressing Human Mutant alpha-Synuclein.* Int J Mol Sci, 2018. **19**(6).
24. Zhang, L.F., et al., *Resveratrol alleviates motor and cognitive deficits and neuropathology in the A53T alpha-synuclein mouse model of Parkinson's disease.* Food Funct, 2018. **9**(12): p. 6414-6426.
25. Rockenstein, E., et al., *Glucocerebrosidase modulates cognitive and motor activities in murine models of Parkinson's disease.* Hum Mol Genet, 2016. **25**(13): p. 2645-2660.
26. West, C.L., et al., *Squalamine Restores the Function of the Enteric Nervous System in Mouse Models of Parkinson's Disease.* J Parkinsons Dis, 2020. **10**(4): p. 1477-1491.
27. Noorian, A.R., et al., *Alpha-synuclein transgenic mice display age-related slowing of gastrointestinal motility associated with transgene expression in the vagal system.* Neurobiol Dis, 2012. **48**(1): p. 9-19.
28. Tikhonova, M.A., et al., *Evaluating the Effects of Grain of Isogenic Wheat Lines Differing in the Content of Anthocyanins in Mouse Models of Neurodegenerative Disorders.* Nutrients, 2020. **12**(12).
29. Wang, W., et al., *Genomic DNA levels of mutant alpha-synuclein correlate with non-motor symptoms in an A53T Parkinson's disease mouse model.* Neurochem Int, 2018. **114**: p. 71-79.
30. Li, Y., et al., *Sirt1/FoxO1-Associated MAO-A Upregulation Promotes Depressive-Like Behavior in Transgenic Mice Expressing Human A53T alpha-Synuclein.* ACS Chem Neurosci, 2020. **11**(22): p. 3838-3848.

31. Rota, L., et al., *Constipation, deficit in colon contractions and alpha-synuclein inclusions within the colon precede motor abnormalities and neurodegeneration in the central nervous system in a mouse model of alpha-synucleinopathy.* Transl Neurodegener, 2019. **8**: p. 5.
32. Kim, S., et al., *Alpha-synuclein interferes with cAMP/PKA-dependent upregulation of dopamine beta-hydroxylase and is associated with abnormal adaptive responses to immobilization stress.* Exp Neurol, 2014. **252**: p. 63-74.
33. Vidal-Martinez, G., et al., *FTY720/Fingolimod Reduces Synucleinopathy and Improves Gut Motility in A53T Mice: CONTRIBUTIONS OF PRO-BRAIN-DERIVED NEUROTROPHIC FACTOR (PRO-BDNF) AND MATURE BDNF.* J Biol Chem, 2016. **291**(39): p. 20811-21.
34. Huang, Y.R., et al., *Naturally occurring autoantibodies against alpha-synuclein rescues memory and motor deficits and attenuates alpha-synuclein pathology in mouse model of Parkinson's disease.* Neurobiol Dis, 2019. **124**: p. 202-217.
35. Finkelstein, D.I., et al., *Clioquinol Improves Cognitive, Motor Function, and Microanatomy of the Alpha-Synuclein hA53T Transgenic Mice.* ACS Chem Neurosci, 2016. **7**(1): p. 119-29.
36. Oaks, A.W., et al., *Age-dependent effects of A53T alpha-synuclein on behavior and dopaminergic function.* PLoS One, 2013. **8**(4): p. e60378.
37. Sardi, S.P., et al., *Glucosylceramide synthase inhibition alleviates aberrations in synucleinopathy models.* Proc Natl Acad Sci U S A, 2017. **114**(10): p. 2699-2704.
38. Wu, Q., et al., *Chronic mild stress accelerates the progression of Parkinson's disease in A53T α-synuclein transgenic mice.* Exp Neurol, 2016. **285**(Pt A): p. 61-71.
39. Rothman, S.M., et al., *Neuronal expression of familial Parkinson's disease A53T alpha-synuclein causes early motor impairment, reduced anxiety and potential sleep disturbances in mice.* J Parkinsons Dis, 2013. **3**(2): p. 215-29.
40. Tatenhorst, L., et al., *Fasudil attenuates aggregation of alpha-synuclein in models of Parkinson's disease.* Acta Neuropathol Commun, 2016. **4**: p. 39.
41. Hallett, P.J., et al., *alpha-Synuclein overexpressing transgenic mice show internal organ pathology and autonomic deficits.* Neurobiol Dis, 2012. **47**(2): p. 258-67.
42. Yamakado, H., et al., *alpha-Synuclein BAC transgenic mice as a model for Parkinson's disease manifested decreased anxiety-like behavior and hyperlocomotion.* Neurosci Res, 2012. **73**(2): p. 173-7.
43. Mandler, M., et al., *Next-generation active immunization approach for synucleinopathies: implications for Parkinson's disease clinical trials.* Acta Neuropathol, 2014. **127**(6): p. 861-79.
44. Wassouf, Z., et al., *Distinct Stress Response and Altered Striatal Transcriptome in Alpha-Synuclein Overexpressing Mice.* Front Neurosci, 2018. **12**: p. 1033.
45. Subramaniam, S.R., et al., *Chronic nicotine improves cognitive and social impairment in mice overexpressing wild type alpha-synuclein.* Neurobiol Dis, 2018. **117**: p. 170-180.
46. Brown, J.M., et al., *Intranasal Carnosine Mitigates alpha-Synuclein Pathology and Motor Dysfunction in the Thy1-aSyn Mouse Model of Parkinson's Disease.* ACS Chem Neurosci, 2021. **12**(13): p. 2347-2359.
47. Games, D., et al., *Reducing C-terminal-truncated alpha-synuclein by immunotherapy attenuates neurodegeneration and propagation in Parkinson's disease-like models.* J Neurosci, 2014. **34**(28): p. 9441-54.

48. Schwab, K., et al., *A Protein Aggregation Inhibitor, Leuco-Methylthioninium Bis(Hydromethanesulfonate), Decreases alpha-Synuclein Inclusions in a Transgenic Mouse Model of Synucleinopathy.* Front Mol Neurosci, 2017. **10**: p. 447.
49. Wang, L., et al., *Mice overexpressing wild-type human alpha-synuclein display alterations in colonic myenteric ganglia and defecation.* Neurogastroenterol Motil, 2012. **24**(9): p. e425-36.
50. McDowell, K.A., et al., *Sleep dysfunction and EEG alterations in mice overexpressing alpha-synuclein.* J Parkinsons Dis, 2014. **4**(3): p. 531-539.
51. Bermudez, M.L., K.B. Seroogy, and M.B. Genter, *Evaluation of Carnosine Intervention in the Thy1-aSyn Mouse Model of Parkinson's Disease.* Neuroscience, 2019. **411**: p. 270-278.
52. Biju, K.C., et al., *Reduced cerebral blood flow in an alpha-synuclein transgenic mouse model of Parkinson's disease.* J Cereb Blood Flow Metab, 2020. **40**(12): p. 2441-2453.
53. Fleming, S.M., et al., *Olfactory deficits in mice overexpressing human wildtype alpha-synuclein.* Eur J Neurosci, 2008. **28**(2): p. 247-56.
54. Fleming, S.M., et al., *A pilot trial of the microtubule-interacting peptide (NAP) in mice overexpressing alpha-synuclein shows improvement in motor function and reduction of alpha-synuclein inclusions.* Mol Cell Neurosci, 2011. **46**(3): p. 597-606.
55. Fleming, S.M., et al., *Impaired baroreflex function in mice overexpressing alpha-synuclein.* Front Neurol, 2013. **4**: p. 103.
56. Magen, I., et al., *Cognitive deficits in a mouse model of pre-manifest Parkinson's disease.* Eur J Neurosci, 2012. **35**(6): p. 870-82.
57. Torres, E.R.S., et al., *Alpha-synuclein pathology, microgliosis, and parvalbumin neuron loss in the amygdala associated with enhanced fear in the Thy1-aSyn model of Parkinson's disease.* Neurobiol Dis, 2021. **158**: p. 105478.
58. Wang, L., et al., *Abnormal colonic motility in mice overexpressing human wild-type alpha-synuclein.* Neuroreport, 2008. **19**(8): p. 873-6.
59. Li, R., et al., *Piperine promotes autophagy flux by P2RX4 activation in SNCA/alpha-synuclein-induced Parkinson disease model.* Autophagy, 2022. **18**(3): p. 559-575.
60. Grant, L.M., et al., *Vocalization deficits in mice over-expressing alpha-synuclein, a model of pre-manifest Parkinson's disease.* Behav Neurosci, 2014. **128**(2): p. 110-21.
61. Keane, P.C., et al., *Trichloroethylene and its metabolite TaClo lead to degeneration of substantia nigra dopaminergic neurones: Effects in wild type and human A30P mutant alpha-synuclein mice.* Neurosci Lett, 2019. **711**: p. 134437.
62. Gureviciene, I., K. Gurevicius, and H. Tanila, *Aging and alpha-synuclein affect synaptic plasticity in the dentate gyrus.* J Neural Transm (Vienna), 2009. **116**(1): p. 13-22.
63. Marxreiter, F., et al., *Glial A30P alpha-synuclein pathology segregates neurogenesis from anxiety-related behavior in conditional transgenic mice.* Neurobiol Dis, 2013. **59**: p. 38-51.
64. Schell, H., et al., *Impaired c-Fos and polo-like kinase 2 induction in the limbic system of fear-conditioned alpha-synuclein transgenic mice.* PLoS One, 2012. **7**(11): p. e50245.
65. Neuner, J., et al., *A30P alpha-Synuclein interferes with the stable integration of adult-born neurons into the olfactory network.* Sci Rep, 2014. **4**: p. 3931.
66. Freichel, C., et al., *Age-dependent cognitive decline and amygdala pathology in alpha-synuclein transgenic mice.* Neurobiol Aging, 2007. **28**(9): p. 1421-35.

67. Stylianou, M., et al., *Early Disruption of Cortical Sleep-Related Oscillations in a Mouse Model of Dementia With Lewy Bodies (DLB) Expressing Human Mutant (A30P) Alpha-Synuclein.* Front Neurosci, 2020. **14**: p. 579867.
68. Gries, M., et al., *Parkinson mice show functional and molecular changes in the gut long before motoric disease onset.* Mol Neurodegener, 2021. **16**(1): p. 34.
69. Langley, M.R., et al., *Characterization of nonmotor behavioral impairments and their neurochemical mechanisms in the MitoPark mouse model of progressive neurodegeneration in Parkinson's disease.* Exp Neurol, 2021. **341**: p. 113716.
70. Li, X., et al., *Cognitive dysfunction precedes the onset of motor symptoms in the MitoPark mouse model of Parkinson's disease.* PLoS One, 2013. **8**(8): p. e71341.
71. Cong, L., et al., *Multimodal MRI Evaluation of the MitoPark Mouse Model of Parkinson's Disease.* PLoS One, 2016. **11**(3): p. e0151884.
72. Ghaisas, S., et al., *MitoPark transgenic mouse model recapitulates the gastrointestinal dysfunction and gut-microbiome changes of Parkinson's disease.* Neurotoxicology, 2019. **75**: p. 186-199.
73. Fifel, K. and H.M. Cooper, *Loss of dopamine disrupts circadian rhythms in a mouse model of Parkinson's disease.* Neurobiol Dis, 2014. **71**: p. 359-69.
74. Pass, T., et al., *The Impact of Mitochondrial Dysfunction on Dopaminergic Neurons in the Olfactory Bulb and Odor Detection.* Mol Neurobiol, 2020. **57**(9): p. 3646-3657.
75. Langley, M.R., et al., *Manganese exposure exacerbates progressive motor deficits and neurodegeneration in the MitoPark mouse model of Parkinson's disease: Relevance to gene and environment interactions in metal neurotoxicity.* Neurotoxicology, 2018. **64**: p. 240-255.
76. Baumann, A., et al., *Preliminary Evidence of Apathetic-Like Behavior in Aged Vesicular Monoamine Transporter 2 Deficient Mice.* Front Hum Neurosci, 2016. **10**: p. 587.
77. Cui, K., et al., *Restoration of Noradrenergic Function in Parkinson's Disease Model Mice.* ASN Neuro, 2021. **13**: p. 17590914211009730.
78. Taylor, T.N., et al., *Nonmotor symptoms of Parkinson's disease revealed in an animal model with reduced monoamine storage capacity.* J Neurosci, 2009. **29**(25): p. 8103-13.
79. Rabl, R., et al., *Early start of progressive motor deficits in Line 61 alpha-synuclein transgenic mice.* BMC Neurosci, 2017. **18**(1): p. 22.
80. Gabrielyan, L., et al., *Behavioral Deficits and Brain alpha-Synuclein and Phosphorylated Serine-129 alpha-Synuclein in Male and Female Mice Overexpressing Human alpha-Synuclein.* J Alzheimers Dis, 2021. **79**(2): p. 875-893.
81. Goldberg, N.R.S., et al., *Neural Stem Cells Rescue Cognitive and Motor Dysfunction in a Transgenic Model of Dementia with Lewy Bodies through a BDNF-Dependent Mechanism.* Stem Cell Reports, 2015. **5**(5): p. 791-804.
82. Lim, J., et al., *LRRK2 G2019S Induces Anxiety/Depression-like Behavior before the Onset of Motor Dysfunction with 5-HT1A Receptor Upregulation in Mice.* J Neurosci, 2018. **38**(7): p. 1611-1621.
83. Matikainen-Ankney, B.A., et al., *Parkinson's Disease-Linked LRRK2-G2019S Mutation Alters Synaptic Plasticity and Promotes Resilience to Chronic Social Stress in Young Adulthood.* J Neurosci, 2018. **38**(45): p. 9700-9711.
84. Volta, M., et al., *Chronic and acute LRRK2 silencing has no long-term behavioral effects, whereas wild-type and mutant LRRK2 overexpression induce motor and cognitive deficits and altered regulation of dopamine release.* Parkinsonism Relat Disord, 2015. **21**(10): p. 1156-63.

85. Crown, L.M., et al., *Sleep Spindles and Fragmented Sleep as Prodromal Markers in a Preclinical Model of LRRK2-G2019S Parkinson's Disease.* Front Neurol, 2020. **11**: p. 324.
86. Adeosun, S.O., et al., *Human LRRK2 G2019S mutation represses post-synaptic protein PSD95 and causes cognitive impairment in transgenic mice.* Neurobiol Learn Mem, 2017. **142**(Pt B): p. 182-189.
87. Glasl, L., et al., *Pink1-deficiency in mice impairs gait, olfaction and serotonergic innervation of the olfactory bulb.* Exp Neurol, 2012. **235**(1): p. 214-27.
88. Maynard, M.E., et al., *Loss of PTEN-induced kinase 1 (Pink1) reduces hippocampal tyrosine hydroxylase and impairs learning and memory.* Exp Neurol, 2020. **323**: p. 113081.
89. Agnihotri, S.K., et al., *PINK1 deficiency is associated with increased deficits of adult hippocampal neurogenesis and lowers the threshold for stress-induced depression in mice.* Behav Brain Res, 2019. **363**: p. 161-172.
90. Ferris, C.F., et al., *Evidence of Neurobiological Changes in the Presymptomatic PINK1 Knockout Rat.* J Parkinsons Dis, 2018. **8**(2): p. 281-301.
91. Hoffmeister, J.D., C.A. Kelm-Nelson, and M.R. Ciucci, *Quantification of brainstem norepinephrine relative to vocal impairment and anxiety in the Pink1-/- rat model of Parkinson disease.* Behav Brain Res, 2021. **414**: p. 113514.
92. Marquis, J.M., S.E. Lettenberger, and C.A. Kelm-Nelson, *Early-onset Parkinsonian behaviors in female Pink1-/- rats.* Behav Brain Res, 2020. **377**: p. 112175.
93. Billia, F., et al., *PTEN-inducible kinase 1 (PINK1)/Park6 is indispensable for normal heart function.* Proc Natl Acad Sci U S A, 2011. **108**(23): p. 9572-7.
94. Beauchamp, L.C., et al., *Ablation of tau causes an olfactory deficit in a murine model of Parkinson's disease.* Acta Neuropathol Commun, 2018. **6**(1): p. 57.
95. Li, Z., et al., *Seizure resistance without parkinsonism in aged mice after tau reduction.* Neurobiol Aging, 2014. **35**(11): p. 2617-2624.
96. Lei, P., et al., *Tau deficiency induces parkinsonism with dementia by impairing APP-mediated iron export.* Nat Med, 2012. **18**(2): p. 291-5.
97. Lei, P., et al., *Motor and cognitive deficits in aged tau knockout mice in two background strains.* Mol Neurodegener, 2014. **9**: p. 29.
98. Morris, M., et al., *Age-appropriate cognition and subtle dopamine-independent motor deficits in aged tau knockout mice.* Neurobiol Aging, 2013. **34**(6): p. 1523-9.
99. Dongworth, R.K., et al., *DJ-1 protects against cell death following acute cardiac ischemia-reperfusion injury.* Cell Death Dis, 2014. **5**: p. e1082.
100. Pham, T.T., et al., *DJ-1-deficient mice show less TH-positive neurons in the ventral tegmental area and exhibit non-motoric behavioural impairments.* Genes Brain Behav, 2010. **9**(3): p. 305-17.
101. Li, M., et al., *Impaired D2 receptor-dependent dopaminergic transmission in prefrontal cortex of awake mouse model of Parkinson's disease.* Brain, 2019. **142**(10): p. 3099-3115.
102. Billia, F., et al., *Parkinson-susceptibility gene DJ-1/PARK7 protects the murine heart from oxidative damage in vivo.* Proc Natl Acad Sci U S A, 2013. **110**(15): p. 6085-90.
103. Kyser, T.L., et al., *Characterization of Motor and Non-Motor Behavioral Alterations in the Dj-1 (PARK7) Knockout Rat.* J Mol Neurosci, 2019. **69**(2): p. 298-311.
104. Liu, Q., et al., *An unexpected improvement in spatial learning and memory ability in alpha-synuclein A53T transgenic mice.* J Neural Transm (Vienna), 2018. **125**(2): p. 203-210.

105. Uemura, N., et al., *alpha-Synuclein Spread from Olfactory Bulb Causes Hyposmia, Anxiety, and Memory Loss in BAC-SNCA Mice.* Mov Disord, 2021. **36**(9): p. 2036-2047.
106. Hamill, R.W., et al., *Autonomic dysfunction and plasticity in micturition reflexes in human alpha-synuclein mice.* Dev Neurobiol, 2012. **72**(6): p. 918-36.
107. La Vitola, P., et al., *Peripheral inflammation exacerbates alpha-synuclein toxicity and neuropathology in Parkinson's models.* Neuropathol Appl Neurobiol, 2021. **47**(1): p. 43-60.
108. Tikhonova, M.A., et al., *Effects of Grape Polyphenols on the Life Span and Neuroinflammatory Alterations Related to Neurodegenerative Parkinson Disease-Like Disturbances in Mice.* Molecules, 2020. **25**(22).
109. Giesert, F., et al., *The pathogenic LRRK2 R1441C mutation induces specific deficits modeling the prodromal phase of Parkinson's disease in the mouse.* Neurobiol Dis, 2017. **105**: p. 179-193.
110. Bichler, Z., et al., *Non-motor and motor features in LRRK2 transgenic mice.* PLoS One, 2013. **8**(7): p. e70249.
111. Mikhail, F., et al., *Lack of exacerbation of neurodegeneration in a double transgenic mouse model of mutant LRRK2 and tau.* Hum Mol Genet, 2015. **24**(12): p. 3545-56.
112. Dranka, B.P., et al., *A novel mitochondrially-targeted apocynin derivative prevents hyposmia and loss of motor function in the leucine-rich repeat kinase 2 (LRRK2(R1441G)) transgenic mouse model of Parkinson's disease.* Neurosci Lett, 2014. **583**: p. 159-64.
113. Lopatina, O., et al., *Anxiety- and depression-like behavior in mice lacking the CD157/BST1 gene, a risk factor for Parkinson's disease.* Front Behav Neurosci, 2014. **8**: p. 133.
114. Mizuno, A., et al., *Lipo-oxytocin-1, a Novel Oxytocin Analog Conjugated with Two Palmitoyl Groups, Has Long-Lasting Effects on Anxiety-Related Behavior and Social Avoidance in CD157 Knockout Mice.* Brain Sci, 2015. **5**(1): p. 3-13.
115. Kasai, S., et al., *Selegiline Ameliorates Depression-Like Behavior in Mice Lacking the CD157/BST1 Gene, a Risk Factor for Parkinson's Disease.* Front Behav Neurosci, 2017. **11**: p. 75.
116. Higashida, H., et al., *An immunohistochemical, enzymatic, and behavioral study of CD157/BST-1 as a neuroregulator.* BMC Neurosci, 2017. **18**(1): p. 35.
117. Maekawa, T., et al., *Leucine-Rich Repeat Kinase 2 Is Associated With Activation of the Paraventricular Nucleus of the Hypothalamus and Stress-Related Gastrointestinal Dysmotility.* Front Neurosci, 2019. **13**: p. 905.
118. Beccano-Kelly, D.A., et al., *LRRK2 overexpression alters glutamatergic presynaptic plasticity, striatal dopamine tone, postsynaptic signal transduction, motor activity and memory.* Hum Mol Genet, 2015. **24**(5): p. 1336-49.
119. Maset, A., et al., *Aberrant Patterns of Sensory-Evoked Activity in the Olfactory Bulb of LRRK2 Knockout Mice.* Cells, 2021. **10**(11).
120. Navarro, P., et al., *Memory and exploratory impairment in mice that lack the Park-2 gene and that over-express the human FTDP-17 mutant Tau.* Behav Brain Res, 2008. **189**(2): p. 350-6.
121. Zhu, X.R., et al., *Non-motor behavioural impairments in parkin-deficient mice.* Eur J Neurosci, 2007. **26**(7): p. 1902-11.
122. Rial, D., et al., *Behavioral phenotyping of Parkin-deficient mice: looking for early preclinical features of Parkinson's disease.* PLoS One, 2014. **9**(12): p. e114216.

123. Itier, J.M., et al., *Parkin gene inactivation alters behaviour and dopamine neurotransmission in the mouse.* Hum Mol Genet, 2003. **12**(18): p. 2277-91.
124. Perez, F.A. and R.D. Palmiter, *Parkin-deficient mice are not a robust model of parkinsonism.* Proc Natl Acad Sci U S A, 2005. **102**(6): p. 2174-9.
125. Kubli, D.A., et al., *Parkin protein deficiency exacerbates cardiac injury and reduces survival following myocardial infarction.* J Biol Chem, 2013. **288**(2): p. 915-26.
126. Hollville, E., et al., *Characterization of a Cul9-Parkin double knockout mouse model for Parkinson's disease.* Sci Rep, 2020. **10**(1): p. 16886.
127. Pavia-Collado, R., et al., *Intracerebral Administration of a Ligand-ASO Conjugate Selectively Reduces alpha-Synuclein Accumulation in Monoamine Neurons of Double Mutant Human A30P*A53T*alpha-Synuclein Transgenic Mice.* Int J Mol Sci, 2021. **22**(6).
128. Lelan, F., et al., *Effects of Human Alpha-Synuclein A53T-A30P Mutations on SVZ and Local Olfactory Bulb Cell Proliferation in a Transgenic Rat Model of Parkinson Disease.* Parkinsons Dis, 2011. **2011**: p. 987084.
129. Ikuno, M., et al., *GBA haploinsufficiency accelerates alpha-synuclein pathology with altered lipid metabolism in a prodromal model of Parkinson's disease.* Hum Mol Genet, 2019. **28**(11): p. 1894-1904.
130. Hosford, P.S., et al., *Synuclein Deficiency Results in Age-Related Respiratory and Cardiovascular Dysfunctions in Mice.* Brain Sci, 2020. **10**(9).
131. Tian, Y., et al., *Transgenic Mice Expressing Human alpha-Synuclein 1-103 Fragment as a Novel Model of Parkinson's Disease.* Front Aging Neurosci, 2021. **13**: p. 760781.
132. Martinez Hernandez, A., et al., *Low-Expressing Synucleinopathy Mouse Models Based on Oligomer-Forming Mutations and C-Terminal Truncation of alpha-Synuclein.* Front Neurosci, 2021. **15**: p. 643391.
133. Escobar, V.D., et al., *Transgenic mice expressing S129 phosphorylation mutations in alpha-synuclein.* Neurosci Lett, 2014. **563**: p. 96-100.
134. Craven, K.M., et al., *Zinc Exacerbates Tau Pathology in a Tau Mouse Model.* J Alzheimers Dis, 2018. **64**(2): p. 617-630.
135. Lambourne, S.L., et al., *Impairments in impulse control in mice transgenic for the human FTDP-17 tauV337M mutation are exacerbated by age.* Hum Mol Genet, 2007. **16**(14): p. 1708-19.
136. Lambourne, S.L., et al., *Increased tau phosphorylation on mitogen-activated protein kinase consensus sites and cognitive decline in transgenic models for Alzheimer's disease and FTDP-17: evidence for distinct molecular processes underlying tau abnormalities.* Mol Cell Biol, 2005. **25**(1): p. 278-93.
137. Anvret, A., et al., *Adh1 and Adh1/4 knockout mice as possible rodent models for presymptomatic Parkinson's disease.* Behav Brain Res, 2012. **227**(1): p. 252-7.
138. Belin, A.C., et al., *Modeling Parkinson's disease genetics: Altered function of the dopamine system in Adh4 knockout mice.* Behav Brain Res, 2011. **217**(2): p. 439-45.
139. Veenit, V., et al., *The Effect of Early Life Stress on Emotional Behaviors in GPR37KO Mice.* Int J Mol Sci, 2021. **23**(1).
140. Mandillo, S., et al., *Mice lacking the Parkinson's related GPR37/PAEL receptor show non-motor behavioral phenotypes: age and gender effect.* Genes Brain Behav, 2013. **12**(4): p. 465-77.
141. Morgan, R.G., et al., *Relative contributions of severe dopaminergic neuron ablation and dopamine depletion to cognitive impairment.* Exp Neurol, 2015. **271**: p. 205-14.

142. Buhusi, M., et al., *Stress-Induced Executive Dysfunction in GDNF-Deficient Mice, A Mouse Model of Parkinsonism.* Front Behav Neurosci, 2016. **10**: p. 114.
143. Prediger, R.D., et al., *Mice with genetic deletion of the heparin-binding growth factor midkine exhibit early preclinical features of Parkinson's disease.* J Neural Transm (Vienna), 2011. **118**(8): p. 1215-25.
144. Jiang, S., et al., *Alterations of the Motor and Olfactory Functions Related to Parkinson's Disease in Transgenic Mice With a VMAT2-Deficiency in Dopaminergic Neurons.* Front Neurosci, 2020. **14**: p. 356.
145. Sonnier, L., et al., *Progressive loss of dopaminergic neurons in the ventral midbrain of adult mice heterozygote for Engrailed1.* J Neurosci, 2007. **27**(5): p. 1063-71.
146. Wu, G., et al., *Mice deficient in GM1 manifest both motor and non-motor symptoms of Parkinson's disease; successful treatment with synthetic GM1 ganglioside.* Exp Neurol, 2020. **329**: p. 113284.
147. Gil-Tommee, C., et al., *Parkinsonian GM2 synthase knockout mice lacking mature gangliosides develop urinary dysfunction and neurogenic bladder.* Exp Neurol, 2019. **311**: p. 265-273.
148. Parrella, E., et al., *NF-kappaB/c-Rel deficiency causes Parkinson's disease-like prodromal symptoms and progressive pathology in mice.* Transl Neurodegener, 2019. **8**: p. 16.
149. Ageta-Ishihara, N., et al., *Chronic overload of SEPT4, a parkin substrate that aggregates in Parkinson’s disease, causes behavioral alterations but not neurodegeneration in mice.* Mol Brain, 2013. **6**(35).
150. Havrda, M.C., et al., *Behavioral abnormalities and Parkinson's-like histological changes resulting from Id2 inactivation in mice.* Dis Model Mech, 2013. **6**(3): p. 819-27.
